# Enzymological Characterization of Atm, the First Laccase from *Agrobacterium sp*. S5-1, with the Ability to Enhance *In Vitro* digestibility of Maize Straw

**DOI:** 10.1371/journal.pone.0128204

**Published:** 2015-05-26

**Authors:** Wei Si, ZhaoWei Wu, LiangLiang Wang, MingMing Yang, Xin Zhao

**Affiliations:** 1 College of Animal Science and Technology, Northwest A&F University, Yangling, Shaanxi Province, People’s Republic of China; 2 Department of Animal Science, McGill University, Quebec, Canada; Center for Nanosciences and Nanotechnology, MEXICO

## Abstract

Laccase is an enzyme that catalyzes oxidation of phenolic compounds, diamines and aromatic amines. In this study, a novel laccase-like gene (*atm*) in a ligninolyitic isolate *Agrobacterium sp*. S5-1 from soil humus was identified and heterologously expressed in *Escherichia coli*. Atm exhibited its maximal activity at pH 4.5 and at 50°C. This enzyme was tolerant to high temperature, a broad range of pH, heavy metal ions (Co^3+^, Mn^2+^, Cu^2+^ and Ni^2+^, 20 mM) and all tested organic solvents. Furthermore, Atm significantly (*p*<0.05) increased dry matter digestibility of maize straw from 23.44% to 27.96% and from 29.53% to 37.10% after 8 or 24 h of digestion and improved acid detergent fiber digestibility from 5.81% to 10.33% and from 12.80% to 19.07% after 8 or 24 h of digestion, respectively. The combination of Atm and fibrolytic enzymes significantly (*p*<0.05) enhanced neutral detergent fiber digestibility from 19.02% to 24.55% after 24 h of digestion respectively. Results showed treatment with Atm effectively improved *in vitro* digestibility of maize straw, thus suggesting that Atm has an application potential for bioconversion of lignin rich agricultural byproducts into animal feed and cellulosic ethanol.

## Introduction

Laccases (benzenediol/oxygen oxidoreductases, EC 1.10.3.2) are polyphenol oxidases with an application potential in industrial and biotechnological processes such as kraft lignin bleaching, decolorization of recalcitrant dyes and bioremediation of environmental pollutants [1]. Oxidation of aromatic or non-aromatic compounds by laccases is coupled with reduction of dioxygen (O_2_) to water [2]. The active site of lacasses usually contains four copper ions [3]. The signature copper binding regions L1, L2, L3 and L4 exist in all laccases and are used to identify laccases [4].

Laccases are widely distributed among plants, fungi and bacteria. White-rot Basidiomycetes have been characterized as efficient lignin degraders and thus have been an abundant source of laccases [4]. However, lack of functional or efficient expression in heterologous hosts, considerable long cultivate times and a low yield made identification and production of a functional fungal laccase difficult [5]. Databank searches and experimental data have provided evidence for the presence of laccases in prokaryotes [6]. Bacterial laccases may have advantages over classical fungal laccases in terms of applications, due to higher activities and being more stable at high temperatures and high-pH values [7]. For instance, a laccase from *Thermus thermophiles* HB27, the most thermostable laccase ever reported, was resistant to incubation at 85°C for 10 min [8]. In addition, Lbh1 from *Bacillus halodurans* was active at alkaline pH and the laccase activity was stimulated rather than inhibited by chloride, making Lbh1 an interesting biocatalyst in applications for biobleaching [9]. Moreover, a novel CotA-type laccase from *Bacillus pumilus* showed thermo stability and a broad substrate spectrum with a biotechnological potential [5]. Despite the knowledge about widespread occurrence of prokaryotic laccases, fully characterized bacterial laccases are limited in numbers. Therefore, more efforts should be made to discover novel bacterial laccases for recombinant expression, considering their enormous potential for industrial and environmental applications [2].

*Agrobacterium sp*. has been reported to be involved in lignin degradation and decolorization. Sundman demonstrated that the α-conidendrin-decomposing Agrobacterium strains could intensively oxidize ligans isotaxiresinol, iso-olivil and olivil [10]. According to Deschamps et al. (1980), *Agrobacterium sp*. could decompose 35%-42% lignin [11]. In addition, Parshetti et al. (2011) reported that a significant increase in laccase activities was observed during crystal violet decolorization by *Agrobacterium radiobacter* MTCC 8161 [12]. Nevertheless, which enzyme(s) is responsible for lignin degradation in *Agrobacterium sp*. is still unknown.

Lignocellulosic biomass such as maize straw is abundant agricultural by-products. About 250 million tons of maize straw are produced annually in China [13]. Lignin is the limiting factor for use of maize straw as animal feed. Lignified tissues limit feed intake and occupy space in the rumen, which may reduce the attachment of bacteria to digestible substrates. In order to improve the availability of energy potential to ruminal microbes and increase values of agricultural by-products, physical, chemical and biological treatments have been considered for removing of lignin. Among them, biological treatments appear as a feasible and environmentally friendly approach for delignification to increase digestibility. For example, ligninolytic white-rot fungi were selected to improve the nutritive value of different substrates during solid state fermentation aiming to produce a value-added product for ruminants [14]. In addition, Arora and Sharma observed that degradation of lignin by white-rot fungi during solid state fermentation was accompanied by good laccase production and suggested that laccase was responsible for ligninolytic activity [15]. Thus, laccases could have a bioconversion function of lignocellulosic substrates into digestible feed.

In this study, a laccase-like gene (*atm*) was identified from a ligninolytic strain, *Agrobacterium sp*. S5-1, through homologous search and heterologously expressed. The enzymatic properties were investigated, including the optima and stability of temperature and pH, tolerance to enzymatic inhibitors, heavy metal ions and organic solvents. Furthermore, treatment with Atm on ruminal degradability of maize straw *in vitro* was measured to determine its potential for biological applications.

## Materials and Methods

### Ethics statement

The wood decay samples were obtained from a testing site in Mt. Qinling under the ethical approval granted by the Northwest Agriculture and Forestry (A&F) University. No specific permissions were required for these locations/activities. Our study did not involve endangered or protected species.

### Bacterial strains, media and vectors

Soil-borne bacteria were isolated from wood decay samples gathered from Mt. Qinling, Ningshaan, Shaanxi, China. These bacteria were screened for laccase secretion on guaiacol LB plates with guaiacol as a laccase substrate. A strain S5-1, with the highest laccase activity, was selected for this study. It was identified as *Agrobacterium sp*. by 16s rDNA gene sequencing and a phylogenetic analysis (S1 Fig). The sequence of 16s rDNA gene was deposited in the GenBank and has been assigned an accession number KP271103. Cultures were grown and maintained in the Luria-Bertani (LB) medium at 37°C. *Escherichia coli* DH5α (TransGen Biotech, China) was utilized in all cloning procedures. Chaperone Competent Cell pG-KJE8/BL21 and plasmid pCold I DNA (TaKaRa, Japan) were used in all expression processes.

### Molecular cloning of the laccase gene and sequence analysis

Primers Atm-F (5’-GTCATATCACCCTCCCGTTA-3’) and Atm-R (5’- CGAGATTTTCTGAAAACGCT -3’) were designed to amplify laccase-like gene of *Agrobacterium sp*. S5-1 according to the aligned protein sequences of other bacterial and fungal laccases from GenBank. For bioinformatics analyses, other laccase protein sequences from different microbial species were aligned with Atm using the ClustalX program. The conserved domain database was used for predicting and analyzing conserved domains [16].

### Expression and purification of Atm in *E*. *coli*

A laccase-like activity gene was amplified and cloned into pCold I using *Bam*HI and *Hin*dIII restriction sites to construct an expression vector pCold I–*atm*. The vector was transformed into the Chaperone Competent Cell *E*.*coli* pG-KJE8/BL21 by heat-shock. The culture volume for protein expression was 800 ml. Expression of Atm was induced by the addition of 0.5 mg/ml L-arabinose, 5ng/ml tetracycline and IPTG at a final concentration of 0.1 mM at 14°C for 24 h. Copper was not added to the expression medium due to the fact that enzymatic activities were not affected by supplementation of Cu in the expression medium in this study.

All purification steps were carried out on ice. Cells (BL21 with pCold I—*atm*) were disrupted by an Ultrasonic processor. The supernatant containing soluble recombinant protein Atm was collected by centrifugation at 13000 g for 20 min and re-suspended in a binding buffer (20 mM NaH_2_PO_4_, 500 mM NaCl, 15 mM imidazole, pH 7.4). Purification was performed according to the instruction of the HisTrap HP column (GE Healthcare, USA). Fractions containing the purified proteins were pooled and concentrated by Amicon ultrafiltration (membrane cutoff 10 kDa, Millipore, USA). The purity of the enzyme in the eluted fractions was estimated by sodium dodecyl sulfate polyacrylamide gel electrophoresis (SDS-PAGE). The purified recombinant protein was detected by western blot with the anti-(His) 6 mouse monoclonal antibody (Transgen, China).

### Assay for the enzymatic activity and enzymatic properties of Atm

2, 2’-azino-bis-(3-ethylbenzthiazoline-6-sulphonic acid) diammonium salt (ABTS) (Sigma, USA) was employed for the laccase activity assay [17]. All the laccase activity assays were determined in a reaction mixture of 150 μl using ABTS (1 mM) as the substrate in a 100 mM sodium acetate buffer (pH 4.5) at 30°C unless otherwise mentioned. The absorbance coefficient was: ε_420nm_ = 36000 M^-1^ cm^-1^ for ABTS. One unit (U) of the enzymatic activity was defined as 1μmol product generated per minute under 30°C and pH 4.5. The protein concentration was determined using a Bradford Protein Assay Kit (Transgen, China) [18]. All experiments were repeated three times. For the Michaelis-Menten kinetics assay, gradient concentrations of ABTS ranging from 50 μM to 1000 μM were employed and *K*m and *V*max values were analyzed by the GraphPad Prism (Version 5.0) based on Linerweaver-burk plots.

The temperature optimum was measured by performing the laccase activity assay at various temperatures (30°C–70°C) under pH 4.5. The thermo-stability of Atm was investigated by pre-incubation of the enzyme solutions for 10 min to 12 hours in the absence of substrate at pH4.5 at temperatures 30°C, 40°C, 50°C, 60°C, 70°C,and 80°C, respectively before the enzymatic assay. The optimal pH value was determined in 100 mM sodium acetate buffers (pH 2.0–6.0), 100 mM sodium phosphate buffers (6.0–8.0) and a 100 mM glycine—NaOH buffer (pH 9.0). The pH stability was investigated by incubating the enzyme over a range of pH 2.0–9.0 for 1 h at 30°C before the enzymatic assay at a single pH (4.5). Effects of certain metal ions, organic solvents and enzyme inhibitors on enzymatic activities were investigated by preincubation of Atm with the inhibitors at 30°C for 1 h before the enzymatic assay. These inhibitors included Co^3+^, Cu^2+^, Fe^3+^, Fe^2+^, Mn^2+^, Ni^2+^, Zn^2+^and Ba^2+^ at a final concentration of 20 mM, organic solvents (acetone, dimethyl sulfoxide (DMSO), methanol, ethanol at a final volume fraction of 10% or 30%), sodium dodecyl sulfate (SDS, final concentrations: 5 mM and 2 mM), ethylene diamine tetraacetie acid (EDTA, final concentrations: 5 mM and 20 mM), dithiothreitol (DTT, final concentrations: 5 mM and 2 mM) and sodium azide (final concentrations: 5 mM and 2 mM).

### Treatment with Atm on degradability of maize straw

The maize straw was milled to pass 30 mesh screen. The retained maize straw was used for the experiment and contained 26.0% cellulose, 24.8% hemicellulose, 13.7% lignin and 35.5% others. Atm (200 mg) was dissolved in 10 ml of double-distilled water and was sprayed onto 40-g maize straw. Control samples were sprayed with water alone. After 12 hours of incubation at 37°C and 12 hour-air-dry, 2-g samples were transferred into filter bags (pore size small than 25 μm), for later *in vitro* digestibility measurement.

Filter bags with 2-g samples (Atm-treated and water-treated) were put into 250-ml flasks with the addition of fibrolytic enzymes (commercial source) at 0, 0.5 g/100 g dry matter (DM). The fibrolytic enzymes (VTR Bio-Tech Co., Ltd.) contained mainly xylanase and cellulose, with activities of 89.2 U/mg and 48.6 U/mg, respectively. Inoculum consisted of 60 ml fresh rumen fluid and 120 ml pre-warmed (39°C) artificial saliva [19]. Rumen fluid was filtered through four-layered muslin cloth. After flushing with CO_2_ gas, the bottles were kept at 39°C for 8 and 24 h. The weight loss in dry matter during the incubation corresponded to 8 and 24h *in vitro* DM digestibility (IVDMD). Neutral detergent fiber (NDF) and ADF analyses were determined according to Van Soest et al. [20]. In addition, for experiments comparing treatments, a one-way analysis of variance (ANOVA) with the least significant difference test was utilized. Results were considered significant if P values were <0.05.

## Results and Discussion

### Gene cloning, expression and purification of a recombinant laccase Atm

**Fig 1:**
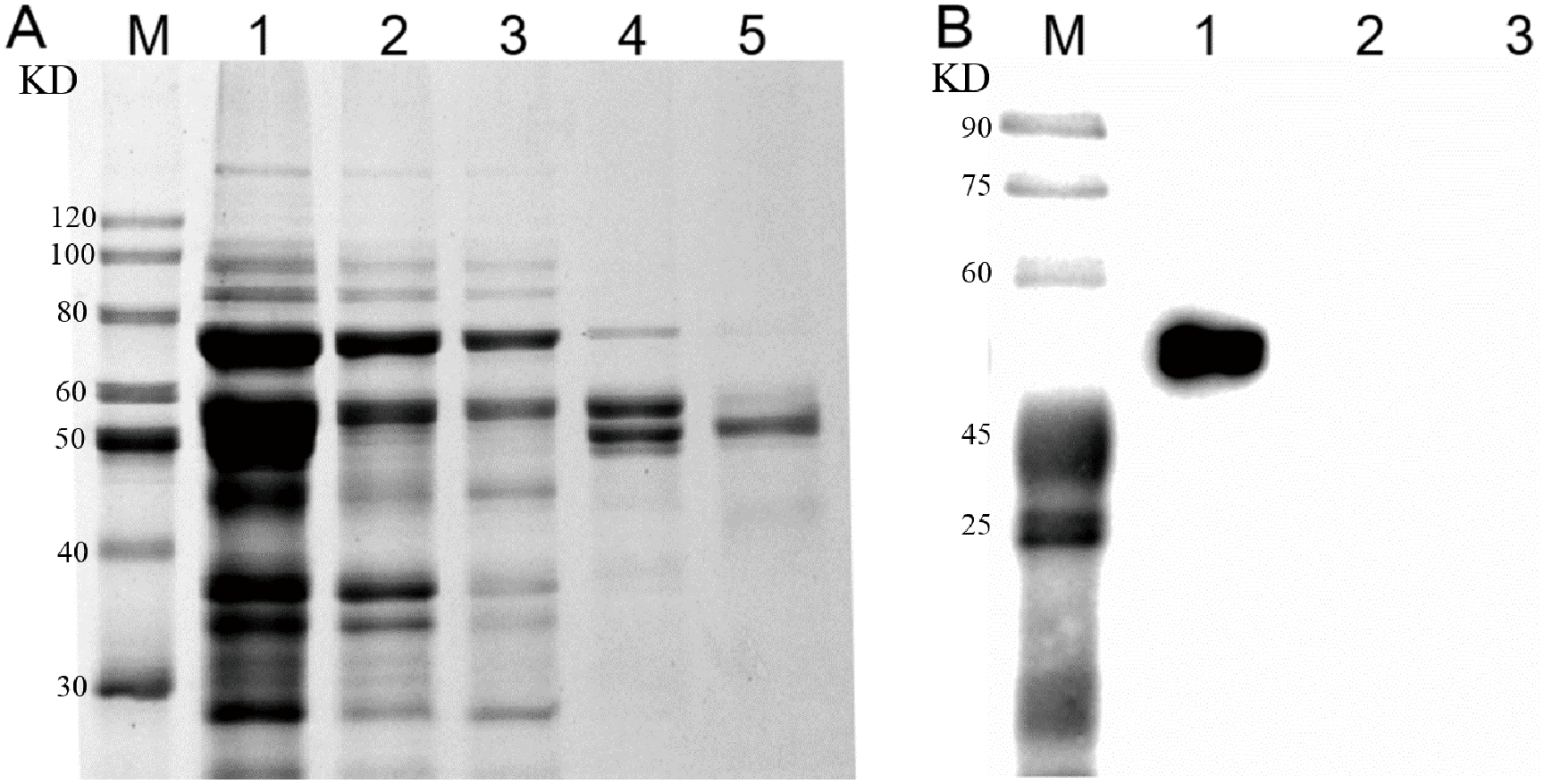
SDS-PAGE and Western blot analysis of Atm. A. SDS-PAGE analysis. M: Protein markers; Lane 1: cell extract; Lane 2: unbound proteins in the flow through from the column; Lane 3: the fraction from the wash buffer containing 40 mM imidazole; Lane 4: the fraction from the wash buffer containing 100 mM imidazole; Lane 5: the fraction from the elution buffer containing 500 mM imidazole. B. Western blot analysis. M: Protein markers; lane 1: protein extracts from pG-KJE8/BL21 with pCold I—atm; lane 2: protein extracts from pG-KJE8/BL21 with pCold I, as a negative control; lane 3, protein extracts from pG-KJE8/BL21, as an additional negative control.

A 1311bp ORF (which has been deposited in the GenBank with an accession No.KP271102) was amplified from the genome of *Agrobacterium sp*. isolate S5-1 and was designated as *atm*. It encoded a protein with 437 amino acids and a molecular mass of 48.57 KDa. Atm was expressed in *E*. *coli* and purified using the nickel affinity chromatography (Fig 1A). To the best of our knowledge, this is the first full-length laccase from *Agrobacterium sp*. The molecular mass of the purified protein was approximately 50 KDa by western blot analysis (Fig 1B). Activities were low in the absence of Cu^2+^, so 1mM Cu^2+^ was added into the assay buffer. The purified laccase activity was 185.4 U mg^-1^ with ABTS as the substrate. According to the Linerweaver-burk plots, *K*_m_ and *k*_cat_ values were 230.8 μM and 32.8 s^-1^, respectively. The hyperthermophilic laccase from *Thermus thermophilus* HB27 had a *K*m of 900 μM and a *k*cat of 24.6 s^-1^ for ABTS. On the other hand, CotA from *B*. *licheniformis* had a *K*m of 6.5 μM and a *k*cat of 83 s^-1^ for ABTS [8, 21].

### Characterization of Atm as a laccase

**Fig 2:**
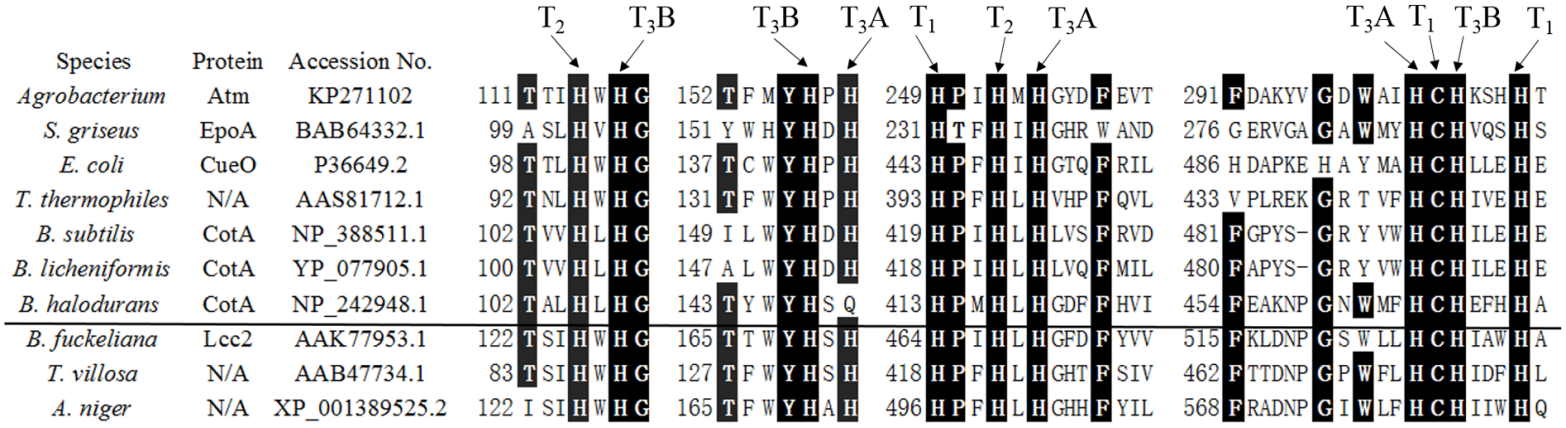
Alignment of the four copper-binding sites in laccases-related proteins. Conserved amino acids are highlight in black. T_1_, T_2_, T_3_A, and T_3_B indicate the putative corresponding type 1, 2, and 3 copper centers. For each copper center, arrows point to the copper-binding amino acid residues. The horizontal line separates bacterial proteins (above the line) from fungal proteins (below the line). *S*. *griseus*: *Streptomyces griseus*; *E*. *coli*: *Escherichia coli* K-12; *T*. *thermophilus*: *Thermus thermophilus* HB27; *B*. *subtilis*: *Bacillus subtilis*; *B*. *licheniformis*: *Bacillus licheniformis*; *B*. *halodurans*: *Bacillus halodurans* C-125; *B*. *fuckeliana*: *Botryotinia fuckeliana*; *T*. *villosa*: *Trametes villosa*; *A*. *niger*: *Aspergillus niger*.

In order to analyze the phylogenetic relationship between Atm and other published and characterized bacterial laccase proteins [8, 9, 21–24], a multiple sequence alignment was performed using ClustalX (Table 1). The highest identity of Atm with other characterized laccases was found with a laccase from *B*. *halodurans* (NP_242948.1; 20% identity). Bacterial laccases from the same genus showed high identities, as *B*. *licheniformis* was 65% identities with *B*. *subtilis*. However, identities among bacterial laccases from different genera were generally low (less than 32%). Some laccase-like genes and proteins from *Agrobacterium sp*. and several other bacterial species within the family Rhizobiaceae were also found through the bioinformatic analysis but not characterized [25]. As shown in S1 Table, the identities among Atm and laccases from *Agrobacterium sp*. were more than 80%. On the other hand, the identities with the laccases from *Bradyrhizobium sp*., *Sinorhizobium sp*. and *Rhizobium sp*. within the Rhizobiaceae family were about 60%, less than 40% and about 30%, respectively. Our results were in general agreement with a previous publication that the overall similarities between bacterial laccases were rather low [8]. This phenomenon might be attributed to different biological functions of bacterial laccases. Previous studies showed that CotA from *B*. *subtilis* might be involved in formation of pigments and resist to UV light [22], while CueO of *E*.*coli* may respond to excess copper [23]. On the other hand, EpoA of *Streptomyces griseus* may be associated with stimulation of morphogenesis [24]. However, biological functions of Atm from *Agrobacterium sp*. are still unknown. While the overall similarities among bacterial laccases were rather low, the four Histidine-rich copper-binding domains were highly conserved in Atm in comparison with other bacterial and fungal laccases (Fig 2). In addition, Atm exhibited a high activity with ABTS which has been used as a standard substrate for laccase activities [6]. These results confirmed that Atm was a laccase.

**Table 1:** Identity (%) of Atm from *Agrobacterium* (Agr) with other published bacterial laccases as calculated by ClustalX.

Sources of enzymes	Identity (%) among Atm and other laccases						
	Agr	Bh	Ec	Tt	Sg	Bl	Bs
Agrobacterium (Agr)		20	12	17	19	11	13
*B*. *halodurans* (Bh)			16	21	18	18	19
*E*. *coli* (Ec)				32	15	25	21
*T*. *thermophiles* (Tt)					11	18	25
*S*. *griseus* (Sg)						13	13
*B*. *licheniformis* (Bl)							65
*B*. *subtilis* (Bs)							

Atm with 436 residues is considered as a two-domain laccase (S2 Fig), which may lack the first of the three domains compared to the traditional three-domain fungal and bacterial lacasses [26, 27]. The two-domain laccases, which have only been identified in prokaryotes so far, also contain four copper ions similar to those of classical three-domain laccases. SLAC from *Streptomyces coelicolor* (343 aa, CAB45586) and EpoA from *Streptomyces griseus* (348 aa, BAB64332) belong to type [B] laccases, while mgLAC (359aa, AB469330) belongs to type [C] laccases [25–27]. A phylogenetic tree of these two-domain laccases and representative three-domain laccases is shown in S2 Fig. Based on the S2 Fig, Atm is a type [B] laccase.

### Enzymatic properties of Atm

**Fig 3:**
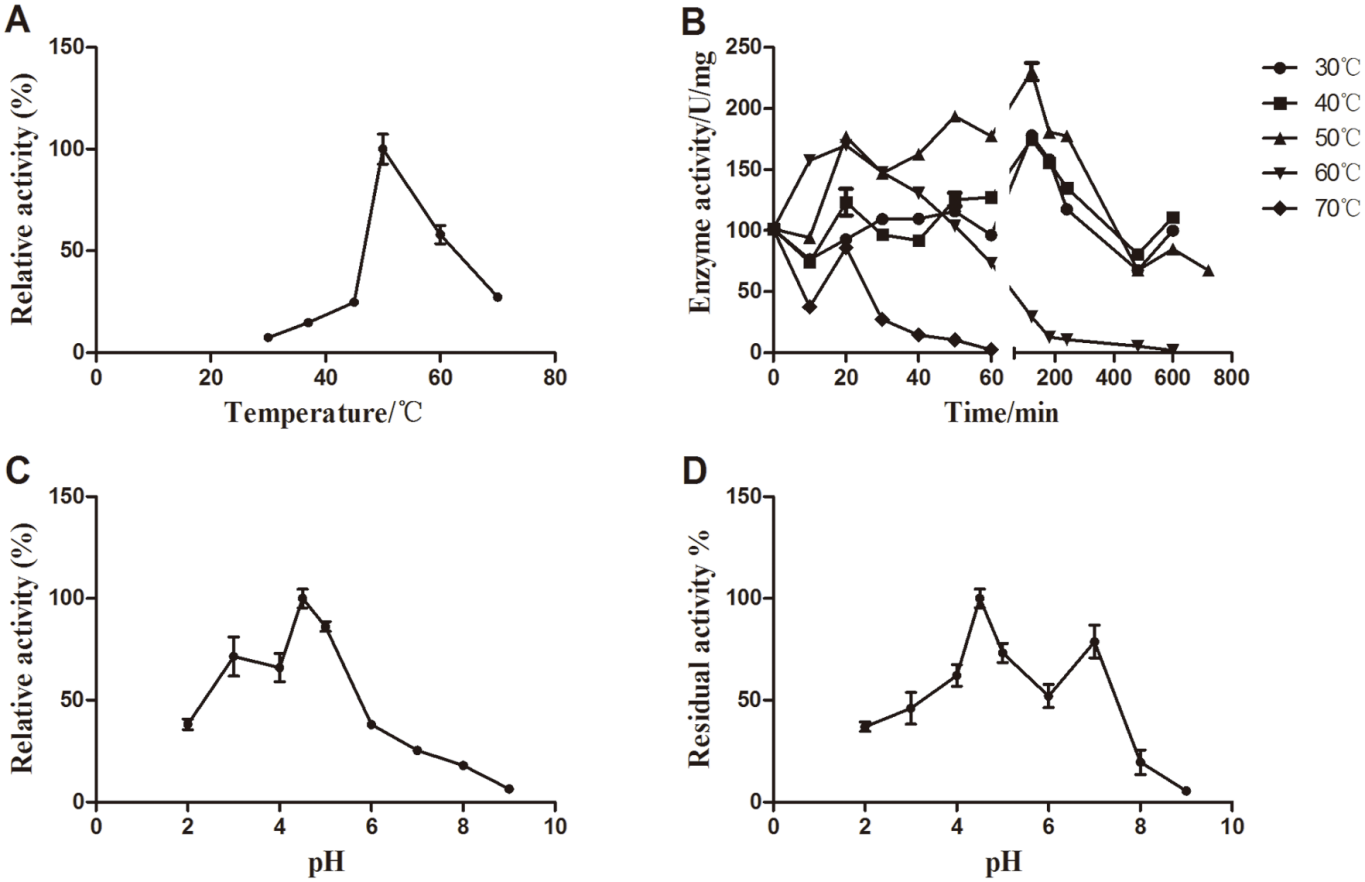
Optimal temperature, thermal tolerance, optimal pH and pH stability of Atm. A. The optimal temperature was determined at various temperatures (30°C–70°C) and at pH 4.5. B. The thermos-stability of Atm was investigated by pre-incubation of the enzyme solutions for 10 min to 12 hours in the absence of substrate at pH4.5, at different temperatures (30°C–70°C) and residual laccase activities were determined. C. The optimal pH was evaluated at 30°C over a pH range of 2.0–9.0. D. pH stability of Atm was evaluated at 30°C over a pH range of 2.0–9.0. The activity measured at pH 4.5 and at 30°C was considered as 100%. Error bars represent the standard errors of the means.

The purified laccase exhibited its maximal activity at pH 4.5 and at 50°C (Fig 3A and 3C). It was completely inactivated under the incubation above 80°C for more than 10 min. The temperature stability (half-life time t_1/2_) of Atm was only 30 min at 70°C but more than 12 hours at 40°C (Fig 3B). For pH tolerance at 30°C, 40% and 5.5% of enzymatic activities remained after 1 h treatment at pH 2.0 and pH 9.0, respectively (Fig 3D). This laccase retained more than 50% activity in the phosphate-buffered saline at 4°C for one week and in deionized water at 4°C for more than 20 days, suggesting that deionized water was an appropriate storage solvent for Atm while Tris—HCl was the best buffer for the laccase from *γ-proteobacterium* JB [28]. Though the optimal temperature of Atm was 50°C, the enzyme could maintain about 50% residual activity for 30 min at 70°C and more than 12 h at 40°C. Similar to other bacterial laccases [5, 8, 28], Atm showed a very high stability at a broad pH range and high temperatures, indicating that it possessed great biotechnological potential.

**Fig 4:**
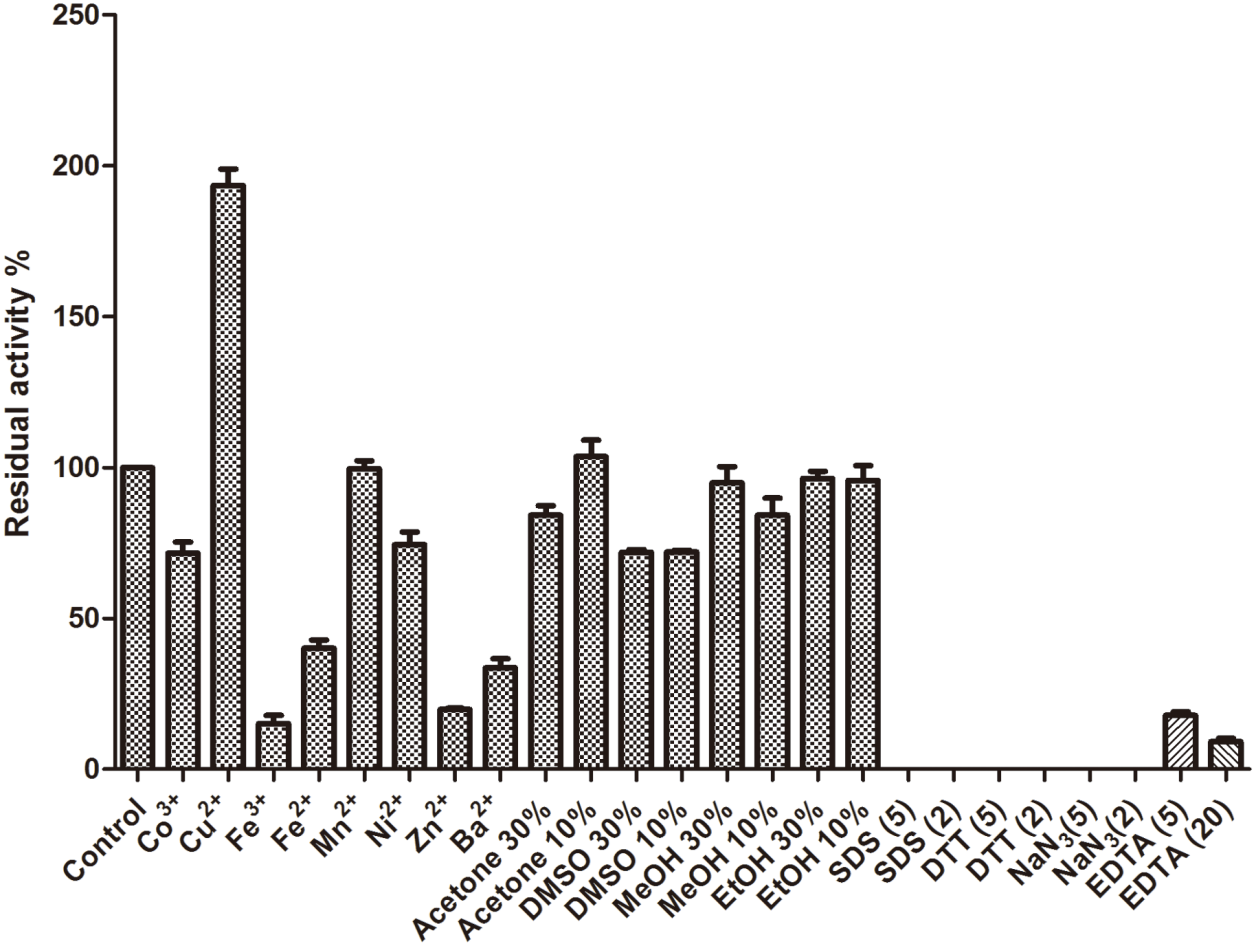
Effect of heavy metals, organic solvents and enzyme inhibitors on enzymatic stability of Atm. DMSO: dimethyl sulfoxide; MeOH: methanol; EtOH: ethanol; SDS: sodium dodecyl sulfate; DTT: dithiothreitol; EDTA: ethylene diamine tetraacetie acid; NaN_3_: sodium azide. Error bars represent the standard errors of the means.

Heavy metal ions, enzyme inhibitors and organic solvents are commonly used in many industrial and agricultural processes and can influence the activity and stability of laccases. As shown in Fig 4, the activity of Atm was markedly improved by the addition of Cu^2+^. Like Atm, *Thermus thermophiles* HB27 laccase needed the presence of Cu^2+^ for its activity [8]. On the other hand, Wu et al. found that the fungal laccase was inhibited by Cu^2+^ even at 0.5 mM and 20 mM Hg^2+^ increased the MAS2 laccase activity [17]. At 20 mM, Co^3+^ and Ni^2+^ showed a weak inhibitory effect on the laccase activity, while Zn^2+^, Ba^2+^, Fe^3+^, Fe^2+^ partially inhibited the laccase activity. A previous study showed that Mn^2+^, Ni^2+^, or Zn^2+^ each at 1 mM failed to support the laccase activity perhaps owning to the interaction with the electron transport system of the laccase [8] while only Zn^2+^ partially affected Atm’s activity in this study. Mn^2+^ even at 20 mM did not affect Atm activity. Atm exhibited much better resistance to heavy metals in comparison with other laccases [8, 17], implying that this enzyme could be applied in bioremediation of soil contaminated by heavy metals. EDTA (5 mM and 20 mM) partially inhibited enzyme activities, presumably due to removal of Cu^2+^ in the assay buffer. Atm activity was totally inhibited by DTT, a strong reducing agent on the disulphide bonds, which may suggest the existence of the disulphide structure in its active domain as reported by Wu et al. [17]. Atm activity was also totally inhibited by sodium azide. Johannes and Majcherczyk [29] have suggested that only sodium azide acted as a true laccase inhibitor. SDS, as an enzyme inhibitor, inhibited Atm activity. Atm also exhibited good tolerance to ethanol, methanol, acetone and DMSO, with 84%, 95%, 100% and 72% of the activity remaining at 10%, respectively. The inhibitory effects were not changed significantly (*p*>0.05) with increasing concentrations of ethanol and DMSO from 10% to 30%, suggesting that potential applications of Atm in non-aqueous catalysis. Addition of 30% DMSO led to a significant decrease of a CotA activity, with only 20% of the residue activity [5], suggesting the advantage of Atm over CotA.

### Treatment with Atm on ruminal digestibility

Whether Atm could act on maize straw was next investigated. As shown in Table 2, DM disappearance after 8 h or 24 h digestion was significantly increased from 23.44% to 27.96% and from 29.53% to 37.10% by Atm treatment (*p*<0.05), respectively. Addition of Atm and the fibrolytic enzymes significantly increased *in vitro* DMD from 23.44% to 28.49% after 8 h digestion and NDFD from 19.02% to 24.55% after 24 h digestion, respectively (*p*<0.05). Addition of Atm or the fibrolytic enzymes separately did not improve NDFD. Atm significantly (*p*<0.05) improved ADFD from 5.81% to 10.33% and from 12.80% to 19.07% after 8 or 24 h of digestion, respectively.

**Table 2:** Effects of laccase Atm and/or fibrolytic compound enzymes on ruminal digestibility *in vitro*.

Groups	Enzymes		IVDMD*(%)			
	Atm g/100gDM	MCE* g/100gDM	8h		24h	
			Mean	SEM	Mean	SEM
Control	0	0	23.44^a^	1.30	29.53^a^	0.57
Group 1	1	0	27.96^b^	0.16	37.10^b^	0.23
Group 2	0	1	22.75^a^	1.42	34.76^a^ ^b^	1.25
Group 3	1	1	28.49^b^	0.30	35.52^a^ ^b^	1.25
			NDFD*(%)			
			8h		24h	
			Mean	SEM	Mean	SEM
Control	0	0	10.63^a^	0.38	19.02^a^	1.06
Group 1	1	0	12.92^a^	0.59	21.89^a^	1.35
Group 2	0	1	14.38^a^	1.59	22.66^a^	1.85
Group 3	1	1	14.26^a^	0.38	24.55^b^	0.59
			ADFD*(%)			
			8h		24h	
			Mean	SEM	Mean	SEM
Control	0	0	5.81^a^	0.89	12.80^a^	0.33
Group 1	1	0	10.33^b^	0.37	19.07^b^	1.49
Group 2	0	1	8.72^a^	1.11	13.38 ^a^	2.59
Group 3	1	1	11.28^b^	0.69	16.06 ^a^	0.57

IVDMD*: *in vitro* dry matter digestibility; NDFD*: neutral detergent fiber digestibility; ADFD*: acid detergent fiber digestibility; MCE*: commercial compound enzymes.

^a, b^ Means with different superscripts in the same column differ at P<0.05.

The enhancement of *in vitro* DM digestibility may attribute to lignin degradation as previously described by Arora and Sharma [15]. In this study, Atm significantly (*p*<0.05) increased 8 and 24 h IVDMD and improved NDFD of maize straw in the presence of fibrolytic enzymes while the fibrolytic enzymes alone did not increase IVDMD and NDFD of maize straw, indicating that adding Atm to maize straw could improve the extent of fiber digestion. Treatment with lignocellulolytic enzymes may promote a breakdown of the bonds between lignin and cellulose [30]. Atm, as a putative lignocellulolytic enzyme, may cleave cell wall lignin structure prior to incubation in rumen fluid and consequently expose polysaccharides to fibrolytic enzymes added or from microorganisms in rumen fluid. Xylanse can cleave high molecular weight arabinoxylans to liberate simple molecules freely available to the animals and cellulose can breakdown fibrous part of the cell wall of lignocellulosic biomass [31]. Our explanation was supported by a previous study from Krueger and Adesogan [32]. The authors observed that addition of ferulic acid esterase (FAE), which releases ferulic acid from the cross linkages between ferulic acid and lignin, significantly increased IVDMD and NDFD from mature bahiagrass by cellulose and xylanase. Similar synergistic interaction among ferulic acid esterase, xylanase and cellulose were also reported [33, 34]. The results from our study and others’ also emphasize the importance of synergistic effects of different lignocellulolytic enzymes for bioconversion of lignin rich agricultural byproducts into animal feed and cellulosic ethanol.

## Conclusions

A novel laccase gene (*atm*) from *Agrobacterium sp*. S5-1 was expressed in *E*. *coli* and purified. Atm showed excellent tolerance to high temperature, heavy metal ions and organic solvents under the assay conditions. Furthermore, Atm could significantly (*p*<0.05) increase 8 and 24h IVDMD while the combination of Atm and fibrolytic enzymes could improve 8 h and 24 h NDFD significantly (*p*<0.05). All of these properties demonstrate that Atm has a potential biological application for transforming lignin rich agricultural byproducts into feeds with greater digestibility and higher quality for ruminants.

## Supporting Information

S1 Fig Phylogenetic tree analysis of 16S gene sequences of bacteria which are closely related to strain S5-1. (DOCX)

S2 Fig Phylogenetic analysis of Atm and several other laccases. (DOCX)

S1 Table Identities (%) between Atm and several predicted laccases from Ausec et al.’s study [25]. (DOCX)
