# Supplementary material for: Enzymological Characterization of Atm, the First Laccase from Agrobacterium sp. S5-1, with the Ability to Enhance In Vitro digestibility of Maize Straw
Source: PLoS One. 2015 May 26;10(5):e0128204. doi: 10.1371/journal.pone.0128204 (PMC4444218; doi:10.1371/journal.pone.0128204)

**S1 Fig. Phylogenetic tree analysis of 16S gene sequences of bacteria which are closely related to strain S5-1.**


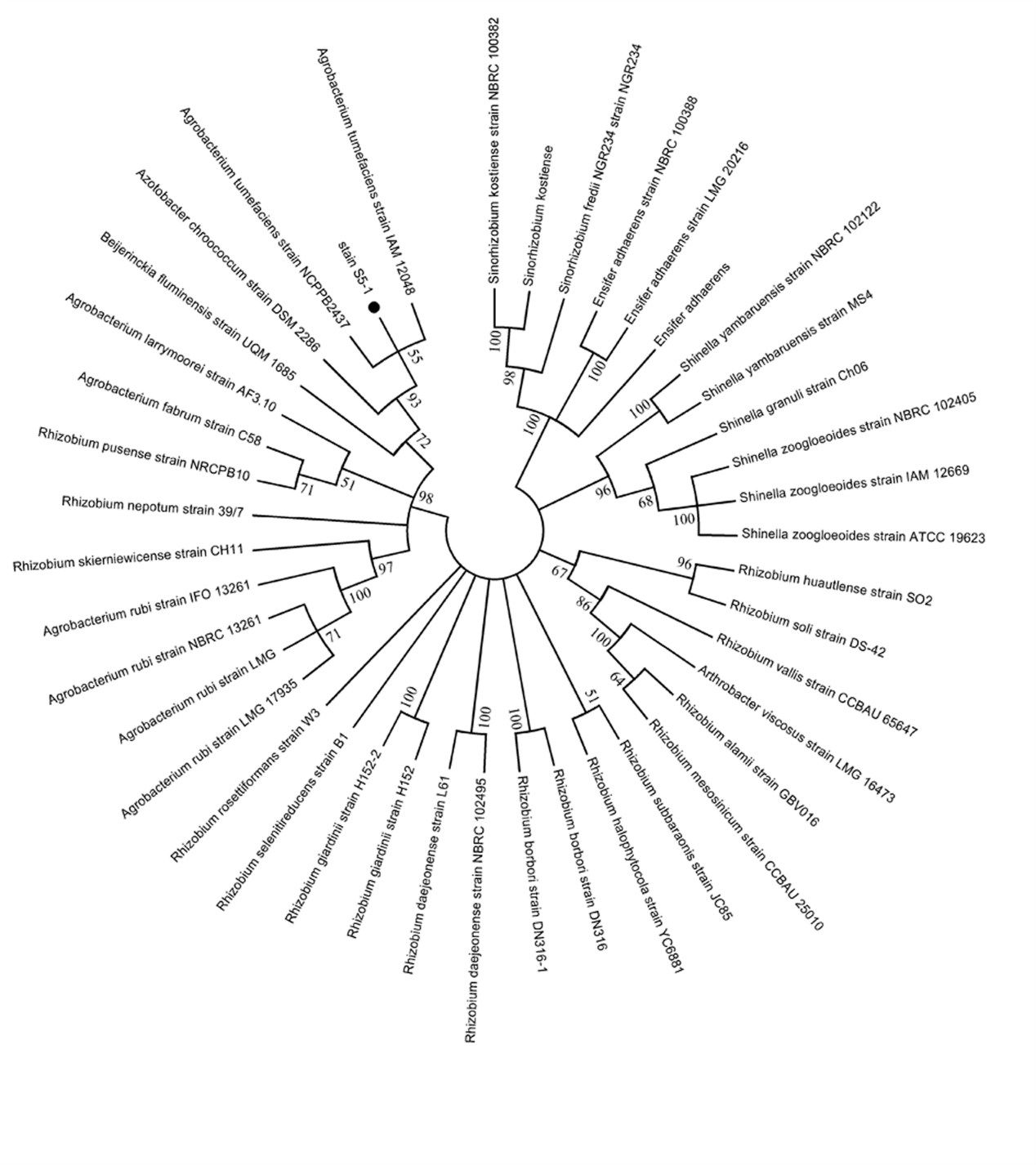

Supplement: S1 Fig — (DOCX) [file pone.0128204.s001.docx]
