# Supplementary material for: Enzymological Characterization of Atm, the First Laccase from Agrobacterium sp. S5-1, with the Ability to Enhance In Vitro digestibility of Maize Straw
Source: PLoS One. 2015 May 26;10(5):e0128204. doi: 10.1371/journal.pone.0128204 (PMC4444218; doi:10.1371/journal.pone.0128204)

**S2 Fig. Phylogenetic analysis of Atm and several other laccases.**

The phylogenetic tree of laccase amino acid sequences was constructed by the MEGA software (version 5.2) using default parameters. Numbers at nodes are levels of bootstrap support (percentages) based on a neighbor-joining analysis of 1,000 resampled datasets by using the Maximum Composite Likelihood method. Type B two-domain laccases from *Agrobacterium tumefaciens* str. C58 (GenBank accession number NP_356650.1), *Streptomyces griseus*, EpoA (GenBank accession number BAB64332.1) and *Streptomyces coelicolor*, SLAC (GenBank accession number CAB45586.1) ; bacterial three-domain laccases from *E.coli*, CueO (GenBank accession number P36649.2), *Thermus thermophiles*(GenBank accession number AAS81712.1), *Bacillus subtilis*, CotA( GenBank accession number NP_388511.1); fungal three-domain laccases from *Trametes villosa* (GenBank accession number AAB47734.1) and *Trametes versicolor*(GenBank accession number XP_008035966.1); mgLAC (GenBank accession number AB469330.1) is a type C two-domain laccase.


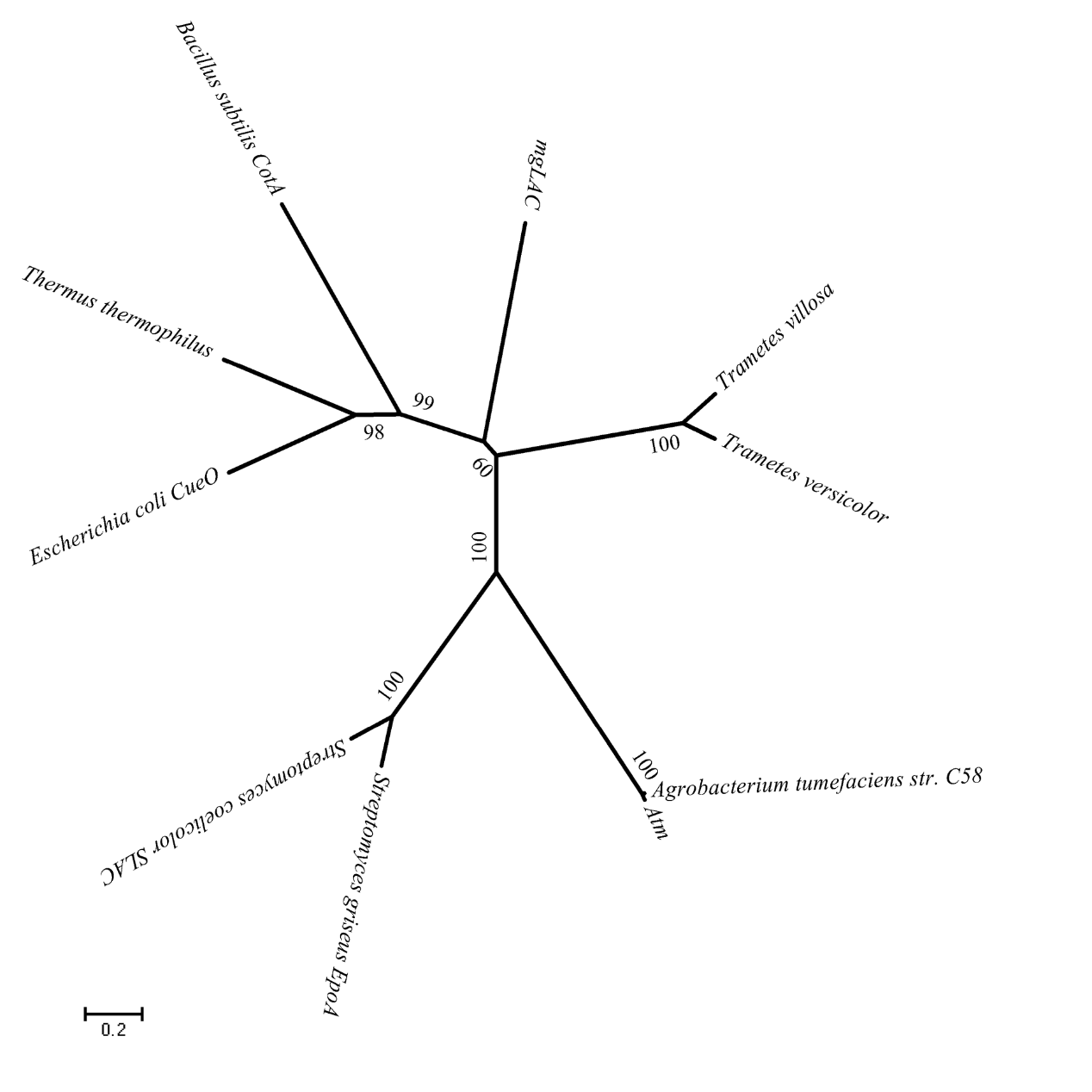

Supplement: S2 Fig — (DOCX) [file pone.0128204.s002.docx]
