# Supplementary material for: Enzymological Characterization of Atm, the First Laccase from Agrobacterium sp. S5-1, with the Ability to Enhance In Vitro digestibility of Maize Straw
Source: PLoS One. 2015 May 26;10(5):e0128204. doi: 10.1371/journal.pone.0128204 (PMC4444218; doi:10.1371/journal.pone.0128204)
Supplement: S1 Table — (DOCX) [file pone.0128204.s003.docx]

**S1 Table. Identities (%) between Atm and several predicted laccases from Ausec et al.’s study [25].**

| **Species** | **Accession number** | **Identities** |
| --- | --- | --- |
| *Agrobacterium vitis* S4 | YP_002546512.1 | 91% |
| *Agrobacterium tumefaciens* str. C58 | NP_356650.1 | 97% |
| *Agrobacterium radiobacter* K84 | YP_002540621.1 | 88% |
| *Bradyrhizobium sp.* BTAi1 | YP_001220465.1 | 66% |
| *Bradyrhizobium sp.* BTAi1 | YP_001242334.1 | 66% |
| *Bradyrhizobium sp.* ORS278 | YP_001203641.1 | 64% |
| *Bradyrhizobium japonicum* USDA 110 | NP_768850.1 | 67% |
| *Sinorhizobium medicae* WSM419 | YP_001314697.1 | 31% |
| *Sinorhizobium meliloti* 1021 | NP_385298.1 | 36% |
| *Sinorhizobium medicae* WSM419 | YP_001326491.1 | 38% |
| *Rhizobium sp.* NGR234 | YP_002822948.1 | 30% |
| *Rhizobium etli* CFN 42 | NP_660002.2 | 30% |
| *Rhizobium sp.* NGR234 | YP_002823642.1 | 29% |
| *Rhizobium etli* Brasil 5 | ZP_03506165.1 | 33% |
